# Extensive deep vein thrombosis treatment using fondaparinux and edoxaban: a case report

**DOI:** 10.1186/s12959-016-0089-x

**Published:** 2016-07-27

**Authors:** Kazuhiro Shimizu, Takeshi Sasaki, Takanobu Tomaru, Hirofumi Noike

**Affiliations:** 1 Department of Internal Medicine, Toho University Sakura Medical Center, Chiba, Japan; 2 Department of Clinical Functional Physiology, Toho University Sakura Medical Center, Chiba, Japan

**Keywords:** Deep vein thrombosis, Edoxaban, Factor Xa inhibitor, Fondaparinux, Venous thromoboembolism

## Abstract

**Background:**

Factor Xa inhibitor is a key drug in the coagulation cascade. Parenteral anticoagulation using low molecular weight heparin or fondaparinux is the recommended form of treatment for most patients presenting with venous thrombosis. Following the acute phase, edoxaban is recommended. We present a case of extensive deep vein thrombosis treated using fondaparinux and edoxaban.

**Case presentation:**

A 63-year-old man with redness, pain, and swelling of the left leg lasting for more than 1 month was referred to our hospital. Ultrasonography revealed a thrombus in the left femoral vein. Computed tomographic angiography revealed clots in the distal right pulmonary artery. Thus, the anticoagulant treatment was initiated with subcutaneous injections of fondaparinux (7.5 mg) for 5 consecutive days, followed by once daily oral administration of edoxaban (60 mg). After 3 months of treatment, a regression of thrombotic clots was shown. Three months later, the remaining clots disappeared, leaving only mural thrombi; no bleeding complications were observed during the treatment period.

**Conclusion:**

The anticoagulant treatment with subcutaneous fondaparinux and subsequently with oral edoxaban was effective for treating extensive deep vein thrombosis.

**Electronic supplementary material:**

The online version of this article (doi:10.1186/s12959-016-0089-x) contains supplementary material, which is available to authorized users.

## Background

Conventional bridging therapy with unfractionated or low-molecular weight heparin followed by vitamin K antagonist (VKA) is widely used for treating venous thromboembolism (VTE). However, VKA requires laboratory monitoring and dose adjustment, which is often difficult、even for specialist physicians [1], and may be complicated by drug or food interactions. Recently, edoxaban was developed as a direct inhibitor of factor Xa. A rapid onset of treatment effect was reported in patients with deep vein thrombosis (DVT) [2–4]. Edoxaban does not share the limitations of VKA and only a few drug interactions have been observed. In an epidemiological study conducted by VTE specialists, the Japan VTE Treatment Registry [5] reported that unfractionated heparin control was insufficient in 40 % of the patients. In Western countries, unfractionated heparin has been replaced with fixed-dose low-molecular weight heparin or fondaparinux, and ambulatory treatment has been more frequently performed than in-hospital treatment since 2011 [6].

We report the experience of a patient with extensive DVT in his left leg, who received successful the anticoagulant treatment with subcutaneous fondaparinux and oral edoxaban. In particular, thrombotic clots gradually regressed without the development of any bleeding complications, leaving only mural thrombi after 6 months of treatment.

## Case presentation

A 63-year-old man was referred to our hospital because of pain, redness and swelling in his left leg persisting for 1 month. At the onset of symptoms, he consulted the neighboring dermatological clinic where he was diagnosed with cellulitis. Antibiotics and nonsteroidal anti-inflammatory drug were administrated, but his symptoms did not improve. After 1 month, he was referred to our VTE specialist department.

The patient had a history of hypertension, which was treated with a calcium channel blocker and an angiotensin receptor blocker. At the initial examination, his body mass index was 33 kg/m^2^, the vital signs showed a mildly elevated blood pressure (134/84 mmHg) and his peripheral oxygen saturation was 98 % in ambient air. The physical examination revealed a tender and swollen left leg. Laboratory test showed elevated levels of urinary acid (8.3 mg/dL) and blood glucose (116 mg/dL). His HbA1c was 5.7 %. Prothrombin and partial thromboplastin times were normal, although D-dimer concentrations were increased to 5.9 μg/mL (normal, <1.00 μg/mL). Plasma protein C, protein S, and antithrombin levels were within normal limits, and creatinine clearance was 113.8 mL/min, as calculated using the Cockcroft–Gault method. Chest X-rays showed no abnormalities. Echocardiography was significant for mild concentric left ventricular hypertrophy with normal left ventricular function and showed no signs of increased right ventricular pressure.

**Fig. 1:**
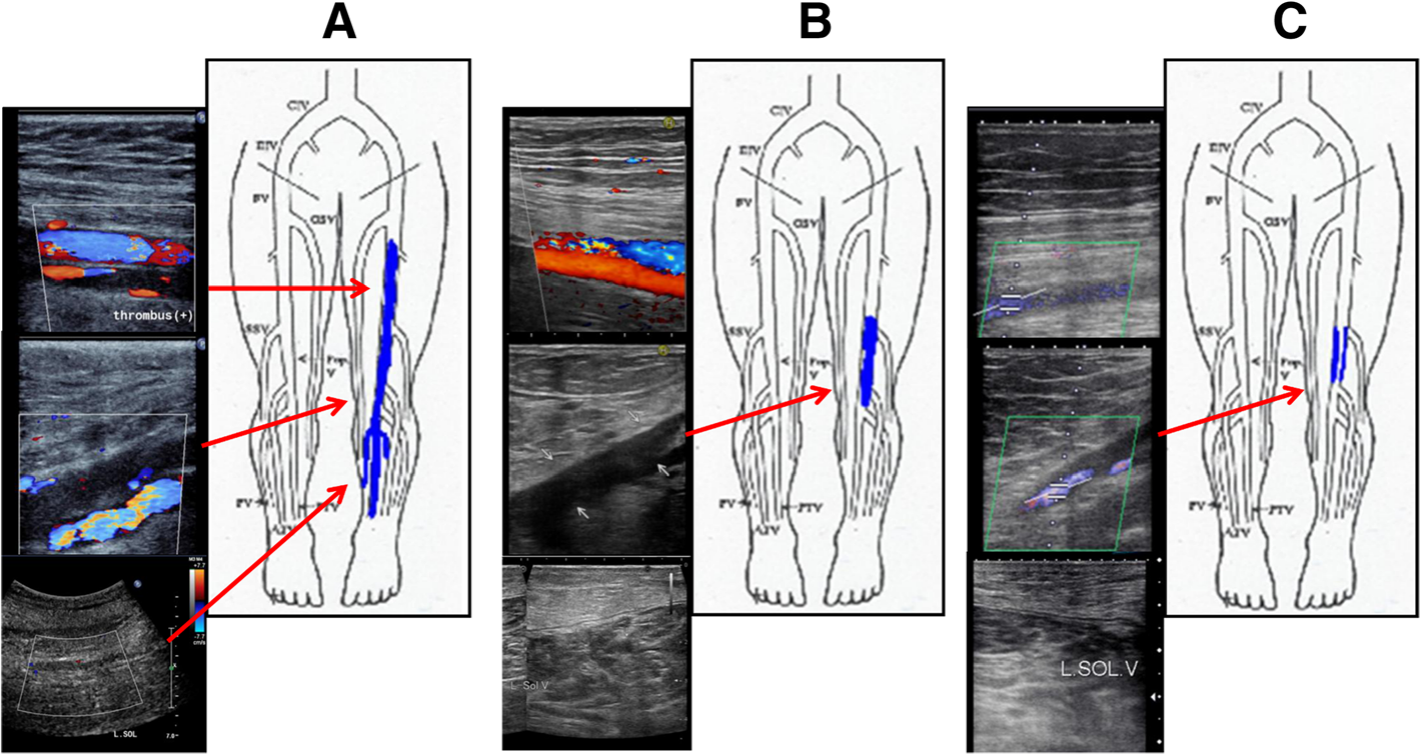
Ultrasonography showing the regression of deep venous thrombosis. **a** Ultrasonography (iU-22, Philips) images of the left lower extremity showing extensive deep vein thrombosis at the initial examination (1 month after the first appearance of symptoms); Additional file 1. **b** Ultrasonography (Noblus, Hitachi Aloka Medical) images of the left lower extremity after 3 months of treatment; Additional file 2. Although some thrombi are still visible in the popliteal vein, no remaining thrombi were observed in the left superficial femoral and soleus muscle veins. **c** Ultrasonography (Aplio XG, Toshiba) images of the left lower extremity after 6 months of treatment; Additional file 3. No remaining thrombi were observed and only mural thrombi remained in the popliteal vein

DVT was suspected because of the patient’s symptoms and elevated D-dimer concentrations. Ultrasonography revealed a thrombus in the left but not in the right femoral vein (Fig. 1), and computed tomographic angiography revealed clots in the right distal pulmonary artery. The maximum circumference of the thighs showed a swelling in the left leg (left calf: 47 cm, left ankle: 28.5 cm, right calf: 44 cm, right ankle: 27 cm).

We instructed the patient to wear medical compression stockings and provided the anticoagulant treatment with subcutaneous injections of fondaparinux (7.5 mg once daily for 5 consecutive days on an outpatient basis), followed by oral administration of edoxaban (60 mg once daily from day 6). Different from western countries, the self- injection of fondaparinux is not allowed in Japan, so the patient was required to visit the hospital every day. After 2 weeks, swelling of the left leg improved, although redness and pain persisted. Subsequent ultrasound examination of the leg after 3 months of treatment with edoxaban revealed no clots in the left superficial femoral vein and soleus muscle vein. Moreover, D-dimer concentrations had dropped to normal levels, indicating decreased activation of secondary fibrinolysis. However, because some clots were still observed in the popliteal vein (Fig. 1), treatment with edoxaban was continued.

After 6 months, the swelling and redness in the lower leg had completely disappeared, and D-dimer concentrations remained normal. Ultrasonography showed further regression of the clots in the left popliteal vein, leaving only mural thrombi (Fig. 1). Throughout the treatment period, doses of fondaparinux and edoxaban were not changed, and no bleeding complications occurred. The symptom of recurrence of pulmonary embolism was not observed during the clinical course. We suggested to end the edoxaban therapy after 6 month, but the patient hoped for a continuation of the anticoagulation therapy.

### Discussion

Until recently in Japan, proper management of DVT required continuous antithrombotic therapy using unfractionated heparin followed by VKA administration within the optimal therapeutic range. In Japan, fondaparinux was approved in 2011. The approval of fondaparinux injections for VTE has relieved patients and physicians of the need for complex dose adjustments, 24-h infusion control, and blood tests every 6 h. Fondaparinux is associated with recurrent VTE and major bleeding rates similar to those occurring with intravenous unfractionated heparin (UFH) [7, 8]. The reasons for outpatient treatment in this case were that the patient was hemodynamically stable and did not suffer from renal failure, cancer, massive pulmonary embolism, heart failure, or bleeding. Furthermore, he had a good understanding about his illness.

Edoxaban was approved in Japan prior to the world as an oral factor Xa inhibitor, and the ESC guidelines recommend the use of edoxaban following acute-phase parenteral anticoagulation therapy. Dabigatran also gained approval as an oral VTE treatment following acute-phase parenteral anticoagulation therapy by Europe and United States besides Japan.

The RE-COVER and Hokusai-VTE trials mandated at least 5 days of heparin or low molecular weight heparin (LMWH) treatment prior to initiation of dabigatran or edoxaban [9–11]. Dabigatran and Edoxaban both showed a no inferiority to warfarin for the prevention of recurrent VTE, and a significantly lower risk of major bleeding. Additionally, a lower proportion of the administered dose of edoxaban is eliminated via the kidneys (35 %) compared to dabigatran (85 %) [12, 13]. The pharmacokinetics of edoxaban were not affected by enoxaparin, whether administered concomitantly or 12 h apart [14]. Edoxaban is a once daily tablet, which is easier to swallow than dabigatran. In addition, dabigatran is not approved for VTE treatment in Japan. These reasons led us to choose this regimen using fondaparinux and edoxaban in this case.

The ACCP guideline stated that in patients with acute DVT of the leg, the guideline suggest early ambulation over initial bed rest (Grade 2C), and that in patients with acute DVT of the leg and whose home circumstances are adequate, the guideline recommend initial treatment at home over treatment in hospital (Grade 1B). Unfortunately this patient couldn’t be hospitalized, so we started the anticoagulant therapy carefully. According to the RIETE registry [15], home treatment of DVT is appropriate in younger men with adequate weight who have no complications of heart failure, respiratory diseases, or cancer. Additionally, we suggest that patients must also understand their condition of illness, visit the hospital daily for fondaparinux administration at least 5 days, and put on and remove medical stockings by themselves or with the help of a family member. Moreover, other medical institutions, including specialist facilities that manage hemorrhagic events or pulmonary embolism, and clinics that cooperate with appropriate specialist institutions are appropriate alternatives to hospitals.

The objectives of DVT treatment include the prevention of recurrence and regression of thrombotic clots, although echogenic masses tend to regress slowly [16]. The precise mechanism by which the factor Xa inhibitor edoxaban causes thrombus regression remains unclear, and no fibrinolytic activity of this agent has been described. Initiation of fibrinolysis occurs through a number of orchestrated interactions among fibrin and plasminogen as well as its activator and results in the generation of plasmin [17]. Therefore, the efficacy of edoxaban may reflect the potency of the fibrinolytic response relative to that of the coagulation response.

Edoxaban reportedly exerts a stable anticoagulant effect compared with conventional drugs [18, 19] and serum D-dimer concentrations are widely used as a marker for secondary fibrinolysis following clot formation. In the present case, D-dimer concentrations rapidly normalized within 2 weeks and remained in the normal range at 6 months. However, clots continued to regress after the normalization of D-dimer concentrations, warranting the development of more sensitive markers of fibrinolysis during slow regression of thrombosis.

In the Hokusai-VTE Study [9], edoxaban was compared with VKA. A therapeutic range was achieved ≥60 % of the treatment period and the reoccurrence of thrombosis was effectively prevented. Factor Xa inhibitors may provide more stable effects on the fibrinolytic system easily.

The ESC guidelines recommend that treatment for DVT be continued for at least 3 months, and extensions of the treatment period should be considered depending on individual patient conditions. Our patient’s VTE risk was idiopathic, which is why we treated him with NOACs for more than 3 months.

In this case, symptoms improved after 3 months, and thrombotic clots remaining in the popliteal vein subsequently regressed further while continuing treatment, indicating the importance of thorough follow-up with ultrasonography and monitoring of subjective symptoms and blood parameters.

Attention is described at the end. In this case, he was misdiagnosed with cellulitis at the onset. The initial misdiagnosis leads to delay of treatment. Cellulitis is an acute, spreading pyogenic inflammation of the dermis and subcutaneous tissue, usually complicating a wound, ulcer, or dermatosis. The area, usually on the leg, is tender, warm, erythematous, and swollen. The differential diagnosis of cellulitis is very important to DVT [20].

## Conclusion

Thrombotic clots gradually regressed during the 6-month anticoagulant treatment with subcutaneous fondaparinux and oral edoxaban. This therapy using fondaparinux and edoxaban would become a new option for DVT treatment.

## Abbreviations

VKA, vitamin K antagonist; VTE, venous thromboembolism; DVT, deep vein thrombosis; UFH, unfractionated heparin; LMWH, low molecular weight heparin; RIETE, Registro Informatizado de la Enfermedad TromboEmbólica

## Additional files

Additional file 1: Ultrasonography (iU-22, Philips) images of the left lower extremity showing extensive deep vein thrombosis at the initial examination. (JPG 45 kb) Additional file 2: Ultrasonography (Noblus, Hitachi Aloka Medical) images of the left lower extremity after 3 months of treatment. (JPG 53 kb) Additional file 3: Ultrasonography (Aplio XG, Toshiba) images of the left lower extremity after 6 months of treatment. (JPG 38 kb)
