# Supplementary figures and images for: Extensive deep vein thrombosis treatment using fondaparinux and edoxaban: a case report
Source: Thromb J. 2016 Jul 27;14:15. doi: 10.1186/s12959-016-0089-x (PMC4962499; doi:10.1186/s12959-016-0089-x)

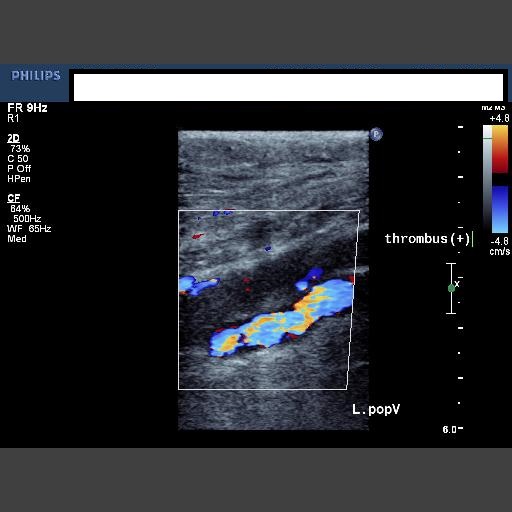

Supplement: Additional file 1: — Ultrasonography (iU-22, Philips) images of the left lower extremity showing extensive deep vein thrombosis at the initial examination. (JPG 45 kb) [file 12959_2016_89_MOESM1_ESM.jpg]

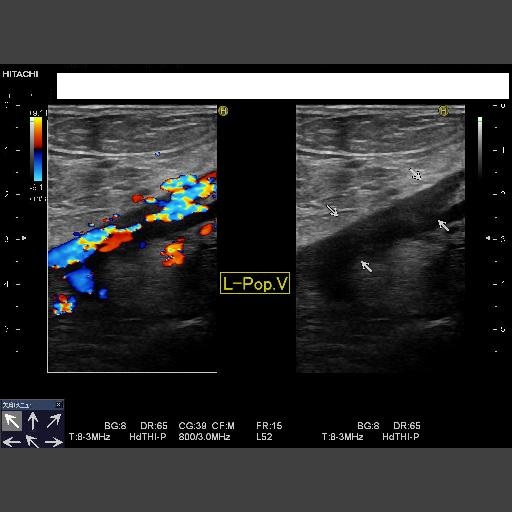

Supplement: Additional file 2: — Ultrasonography (Noblus, Hitachi Aloka Medical) images of the left lower extremity after 3 months of treatment. (JPG 53 kb) [file 12959_2016_89_MOESM2_ESM.jpg]

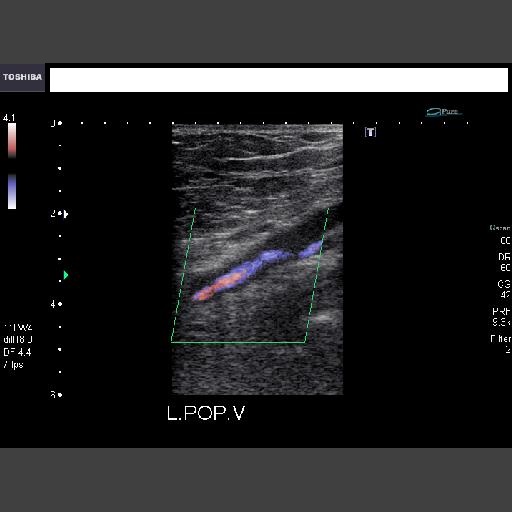

Supplement: Additional file 3: — Ultrasonography (Aplio XG, Toshiba) images of the left lower extremity after 6 months of treatment. (JPG 38 kb) [file 12959_2016_89_MOESM3_ESM.jpg]
